# Airborne Lead (Pb) From Abandoned Mine Waste in Northeastern Oklahoma, USA

**DOI:** 10.1029/2020GH000273

**Published:** 2020-09-04

**Authors:** Junran Li, Julie McDonald‐Gillespie

**Affiliations:** ^1^ Department of Geosciences The University of Tulsa Tulsa OK USA

**Keywords:** metals, health, lake sediment, windborne, particulate matter

## Abstract

Active and abandoned mines pose serious health threats to humans, aquatic, and terrestrial biota. Northeastern Oklahoma, home to a number of Native American Tribes, is part of the well‐known Tri‐State Mining District. More than 100 years of mining production in this area has left numerous, large chat piles in the surrounding environment. Despite numerous studies and efforts on the restoration of metal contamination in this area, no studies have attempted to distinguish the contributions of different sources, particularly from the atmospheric deposition, of metals to the aquatic environment. Here, we analyzed the atmospheric deposition of Pb from Picher, a town surrounded by chat piles, and Tulsa, a primary metropolitan area in northeastern Oklahoma, from 2010 to 2016. We found that chat piles in Picher contain ~20% and 6% of fine particles that are subjective to windborne transport and human inhalation, respectively, and these fine particles contain disproportionally high concentrations of Pb. Despite the absence of industrial and human activities, airborne Pb in Picher is 2–5 times higher than that of Tulsa. A conservative estimate showed that airborne Pb may contribute up to 10% of annual Pb mass flux to a lake 18 km away from the chat piles in Picher and probably a much higher contribution for soil and water located adjacent to Picher. Despite known limitations, our study represents the first attempt to evaluate the significance of Pb‐laden airborne particulate matter from a large‐scale abandoned mining area where the humans are particularly vulnerable to metal exposure.

## Key Points

- The characteristics of airborne Pb associated with a large abandoned mining area in northeastern Oklahoma were examined
- Mining waste contains ~20% and 6% of fine particles that are subjective to windborne transport and human inhalation, respectively
- Airborne Pb in the mining area is 2–5 times higher than Tulsa, Oklahoma, and contributes 10% of Pb in lake sediment close to the mining area

## 1 Introduction

**Figure 1:**
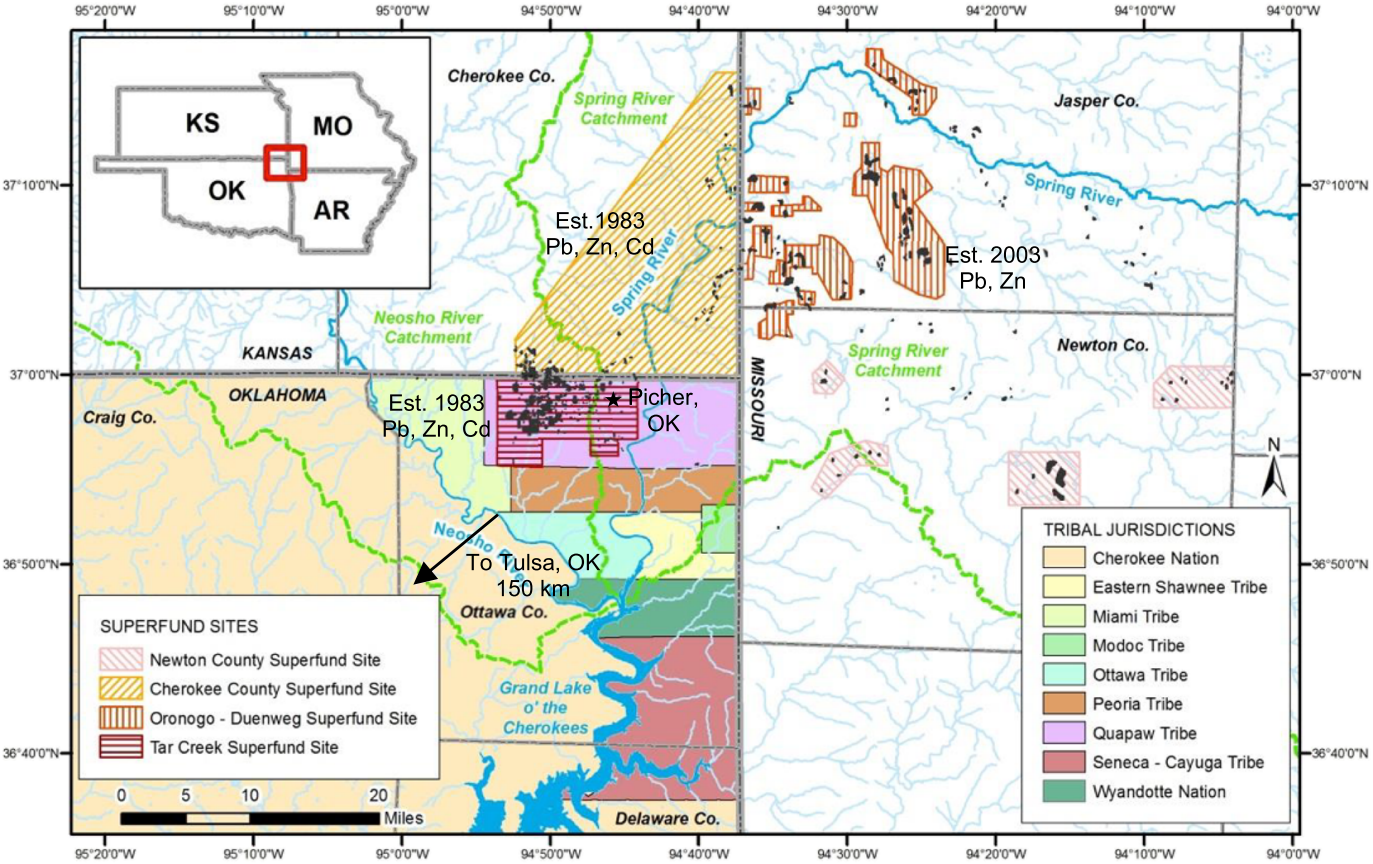
Map of the northeastern Oklahoma and the Tri‐State (Oklahoma, Missouri, and Kansas) mining area. Note that there are nine Native American tribes and three U.S. EPA Superfund sites in the area that were established at different years focusing on different metals. Picher is located within the area of the Tar Creek Superfund Site, which was established in 1983. Modified from Garvin (2016).

Active and abandoned mines are known to cause damage to aquatic and terrestrial biota, air quality, and human health worldwide (Garvin, 2016; Schaider et al., 2007; Zota et al., 2009). In the United States alone, there are more than 557,000 abandoned mines (Hu et al., 2007). One such area is located in northeastern Oklahoma, which is part of a 6,500 km^2^ area known as the Tri‐State Mining District (TSMD) (Figure 1). More than 100 years of lead‐zinc‐cadmium (Pb‐Zn‐Cd) mining production in this area has left numerous, large mine waste piles in the surrounding environment (Hu et al., 2007; Johnson et al., 2016). These mine waste piles, locally termed “chat,” are primarily composed of chert, dolomite, and sulfide minerals (McKnight & Fischer, 1970; United States Environmental Protection Agency, 2016). Studies have shown that the weathering and leaching of the chat piles and the abandoned mine areas serve as sources for metals in food, water, and air particles within and beyond the TSMD (Garvin, 2016; Johnson et al., 2016; Potra et al., 2017; Spruill, 1987; Zota et al., 2009). Due to this contamination, many children in Picher, Oklahoma, have elevated blood Pb levels, which was believed to be related to learning disabilities and other health problems among local children (Oklahoma Department of Environmental Quality, 2016; United States Environmental Protection Agency, 2016).

Analysis on the physical properties of chat showed that the size of the materials varies widely, containing a noticeable proportion of fine particles (Datin & Cates, 2002; Smith et al., 2013). These metal‐containing fine particles may be transported and deposited into a broader environment through mechanisms other than water, for example, wind. Situated close to the Tornado Alley, northeastern Oklahoma and surrounding areas are notorious for variable extreme weather conditions including windstorms and tornados, which may lead to substantial airborne contaminants in this area. While the on‐site monitoring of these events is largely absent, local residents described that on windy days, the fine dust swirls high into the air.

A large body of evidence has shown that exposure to airborne particulate matter (PM) is harmful to human health (e.g., Davidson et al., 2005; Kim et al., 2015; Speizer, 1993). Fine PM such as PM_2.5_, (with aerodynamic diameters less than or equal to 2.5 μm) may cause a range of adverse respiratory and cardiovascular health effects including mortality (Crooks et al., 2016; Krewski et al., 2005; Valavanidis et al., 2008) and heart attacks (Rice & Mittleman, 2017). Exposure to fine airborne particles is also known to be associated with *Coccidioidomycosis*, commonly known as Valley fever in the southwestern United Sates (Centers for Disease Control and Prevention, 2013). Although still under debate, a very recent study reported that atmospheric particulates may act as a virus carrier in the midst of the COVID‐19 virus pandemic in northern Italy (Sterpetti, 2020). Inhalation of metal‐containing PM may be particularly toxic because metals such as Mn, Cd, Zn, and Ni can be transported directly to the brain via olfactory pathways (Zota et al., 2009). Additionally, metal‐containing PM may be deposited in soils, lakes, ponds, and rivers, directly affecting the health of humans, terrestrial, and aquatic biota (Garvin, 2016). A recent study reported that the Pb deposition in topsoil had adverse effects on the cognitive function in preschool‐aged children, particularly boys, in the United States (Clay et al., 2019).

Previous studies show that the average background level of Pb in the sediment or soil in the TSMD area is 17–23 mg/kg (Garvin, 2016; Horowitz, 1991; Pope, 2005). Streambed sediment in the areas contaminated by chat piles, however, had Pb 20 times higher than the background levels (Garvin, 2016; Potra et al., 2017). While numerous efforts have focused on the contamination of metals in soils and waters (e.g., Andrews et al., 2009; Angelo et al., 2007; Beattie et al., 2017; Juracek, 2006), no studies have attempted to distinguish the contributions of different sources of metals, particularly from the atmospheric deposition, to the aquatic environment. The lack of this critical knowledge may hinder the efforts to provide more pertinent management strategies for the tribal human health and welfare and the health of the aquatic biota in the TSMD.

The objective of this study is to characterize the airborne particulate matter and Pb from the chat piles and to estimate the atmospheric input of Pb to the aquatic environment in northeastern Oklahoma, USA. This study took advantage of widely available data sources from multiple federal and state organizations and existing studies. To provide a comparative point view of the characteristics of airborne Pb in northeastern Oklahoma, two locations, Picher, located immediately close to chat piles, and Tulsa, located 150 km southwest of Picher, were investigated (Figure 1). The level of airborne Pb in Tulsa thus represents a benchmark level that is not affected by chat piles and abandoned mines in northeastern Oklahoma, USA.

## 2 Data and Analysis

**Figure 2:**
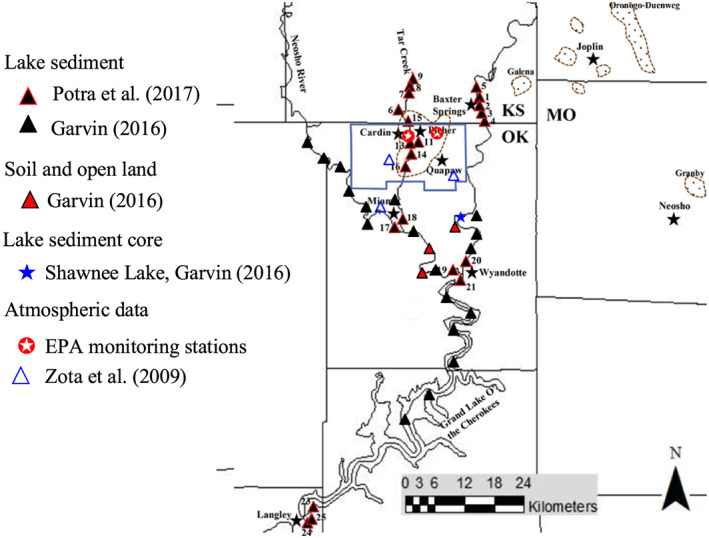
The approximate sampling locations of relevant studies that investigated mining‐related metals in the Tri‐State Mining District. Modified from Potra et al. (2017).

The particle‐size fractions of the chat material and Pb content in different particle‐size ranges were obtained by reanalyzing the data sets of Datin and Cates (2002) and Smith et al. (2013). These data sets contain a large number of samples collected from the chat piles across the Tar Creek Super Fund site, the area with the highest density of chat piles (Figures 1 and 2). Samples in Datin and Cates (2002) were collected from the face of chat piles, and samples were analyzed for Pb using the X‐ray fluorescence in combination with the inductively coupled plasma (ICP) method. For Smith et al. (2013), only the top 5 cm soil data were used in this study, and Pb contents were analyzed by the inductively coupled plasma–mass spectrometry (ICP‐MS) method.

The PM and airborne Pb data sets were acquired from the U.S. Environmental Protection Agency Air Quality System (AQS) database (https://www.epa.gov/outdoor-air-quality-data/interactive-map-air-quality-monitors). The network of the AQS has multiple monitoring stations in northeastern Oklahoma. Only the stations that have continuous monitoring for the period of 2010–2016 were selected for this study. Also, as the town of Picher was officially dissolved in September 2009, direct human impacts on PM were largely excluded thereafter. In Picher, two monitoring stations (36.9857°N, 94.8393°W; 36.9846°N, 94.8249°W) were located in source‐dominated area surrounded by chat piles (Figure 2). The Tulsa site, located in northern Tulsa (36.2049°N, 95.9765°W), was selected to provide a benchmark of atmospheric deposition of Pb outside of the TSMD. The sampling duration for both the Picher and Tulsa sites was 24 hr, and samples were collected every 6 days.

At these monitoring stations, airborne Pb was measured using a high volume total suspended particle (Hi‐Vol TSP) sampler, which applied an airflow rate of approximately 1.4 m^3^/min through 20 cm × 25 cm glass fiber filters. Airborne Pb was extracted from the filters using 3M HNO_3_ and then analyzed by the ICP‐MS method.

The mass flux of airborne Pb was derived from Zota et al. (2009), which monitored the mass of both PM_10_ and PM_2.5_ and their associated Pb content for three locations at different distances from the chat pile source area weekly for a period of 58 weeks (Figure 2). The combined net mass flux of airborne and waterborne Pb was estimated according to Garvin (2016), which analyzed concentrations of Pb for a sediment core collected from a lake located approximately 18 km southeast of Picher. Accordingly, the relative contribution of Pb sources was estimated assuming that the net accumulation of Pb is the result of airborne and waterborne inputs.

The mean values of daily, monthly, and annual airborne Pb were computed for both Picher and Tulsa sites. The daily and monthly PM and airborne Pb were compared with the U.S. National Ambient Air Quality Standards (NAAQS) 24‐hr Primary Standards. A Pearson's correlation analysis was conducted between the monthly airborne Pb and meteorological data for both study sites using *R* software version 3.3.3 (*R* Development Core Team, 2013).

## 3 Results

### 3.1 Pb Content of Chat Pile Material

**Figure 3:**
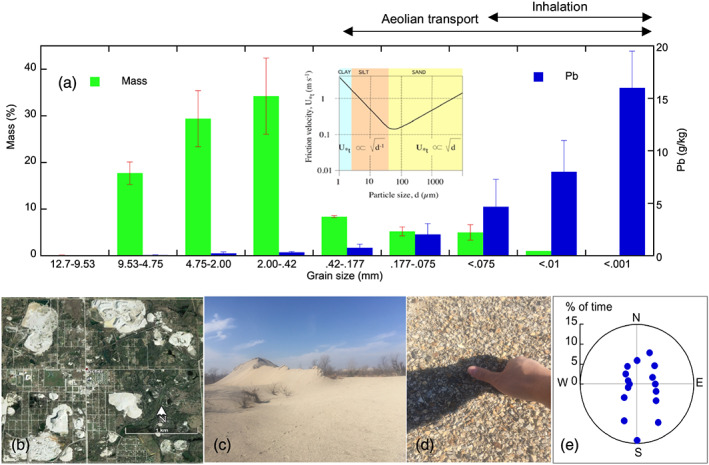
Mass distribution in relation to the content of Pb in various particle‐size fractions in the chat pile material (a), general scenes of the chat (b–d), and the distribution of the primary wind direction in the study area of northeastern Oklahoma (e). The general relationship between particle size and friction velocity of wind erosion is shown in the inset diagram (Shao, 2000). The approximate size range of particles that can be transported by wind and inhaled by human beings is shown on the top right. Data are synthesized from Datin and Cates (2002) and Smith et al. (2013). Error bars are one standard deviation.

Coarse particles with diameters >420 μm account for a majority of the chat pile materials, and particles with diameters <10 μm only composed of 1% of the total mass (Figure 3a). Despite the low mass composition, fine particles contain disproportionally high concentrations of Pb in the chat piles. For instance, coarse particles contain <0.3 g/kg of Pb, whereas fine particles with diameters <10 μm contain 8 g/kg of Pb on average. It is noteworthy that particles that can be transported by wind (diameters <420 μm) accounted for nearly 20% of the total mass, and particles that can be inhaled (diameters <75 μm) constituted 6% of the total mass.

### 3.2 Characteristics of Airborne PM and Pb

**Figure 4:**
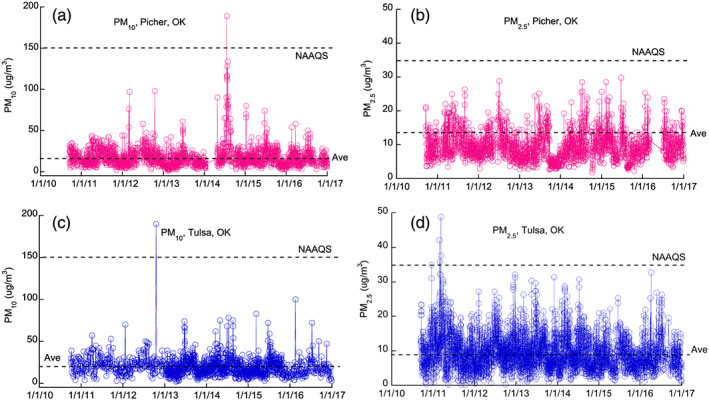
Daily variation of atmospheric PM_10_ and PM_2.5_ monitored at Picher and Tulsa, Oklahoma, during the experimental period of time. The dashed lines show the average concentrations of PM_10_ and PM_2.5_ and the National Ambient Air Quality Standards (NAAQS) 24‐hr primary standards of 150 and 35 μg/m^3^, respectively.

From September 2010 to December 2016, average daily PM_10_ concentrations in both Picher and Tulsa were generally lower than 50 μg/m^3^, and only occasionally went beyond the level of the NAAQS standard of 150 μg/m^3^. Average daily PM_2.5_ concentrations in Tulsa were noticeably higher and more variable than those of Picher, where no case exceeding the NAAQS standard of 35 μg/m^3^ was detected during this period of time (Figure 4).

**Figure 5:**
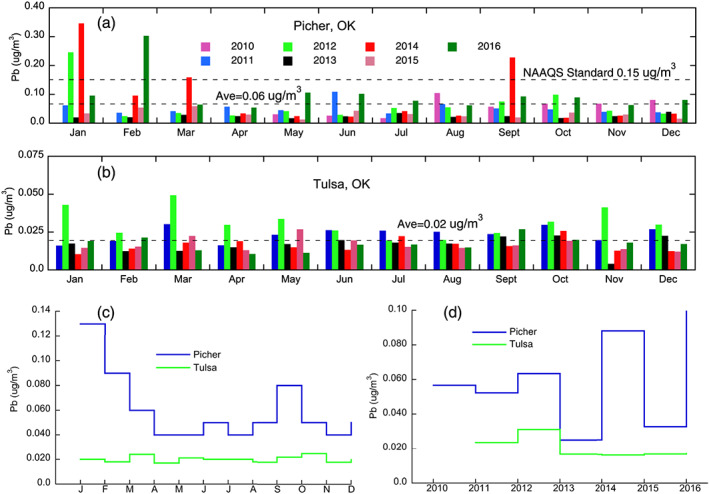
Temporal characteristics of airborne Pb monitored at Picher and Tulsa, Oklahoma, during the experimental period of time.

**Figure 6:**
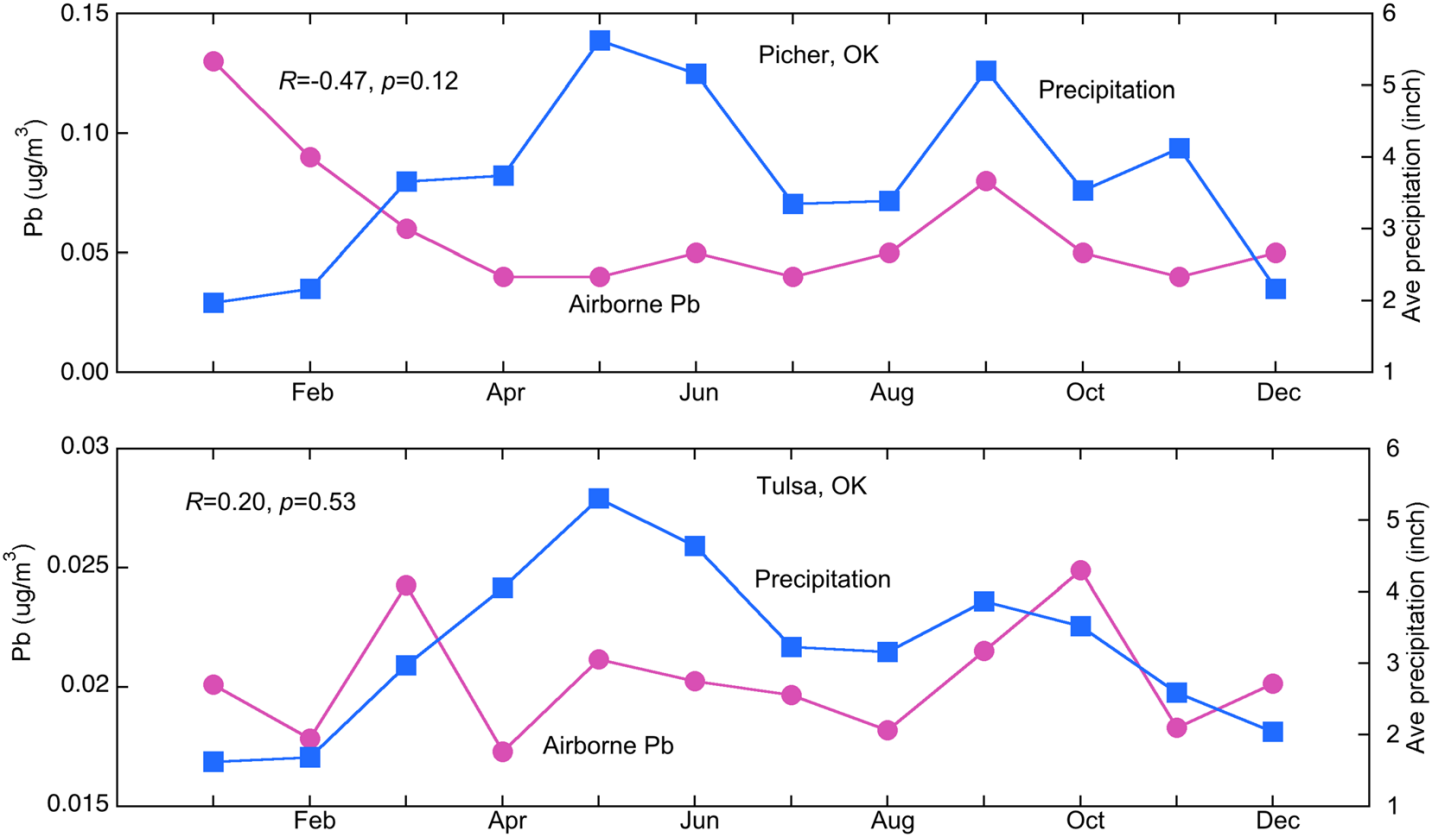
Monthly average airborne Pb and precipitation at Picher and Tulsa, Oklahoma.

Atmospheric Pb in the Picher area has a strong seasonal pattern with two peak times, one in early spring and one in late fall, when the average airborne Pb frequently exceeded 0.15 μg/m^3^, the NAAQS standard for ambient air Pb (Figure 5a). Overall, atmospheric Pb in Tulsa showed small temporal variation with monthly and annual average concentrations noticeably lower than those of Picher (Figures 5b–5d). The Pearson's correlation analysis showed that there is a negative correlation between the monthly airborne Pb and monthly precipitation for the Picher area (*R* = −0.47, *p* = 0.12), and a positive correlation for the Tulsa area (*R* = 0.20, *p* = 0.53), and neither is significant at the level of *p* < 0.05 (Figure 6).

### 3.3 Estimated Contribution of Airborne Pb to the Aquatic Environment

**Figure 7:**
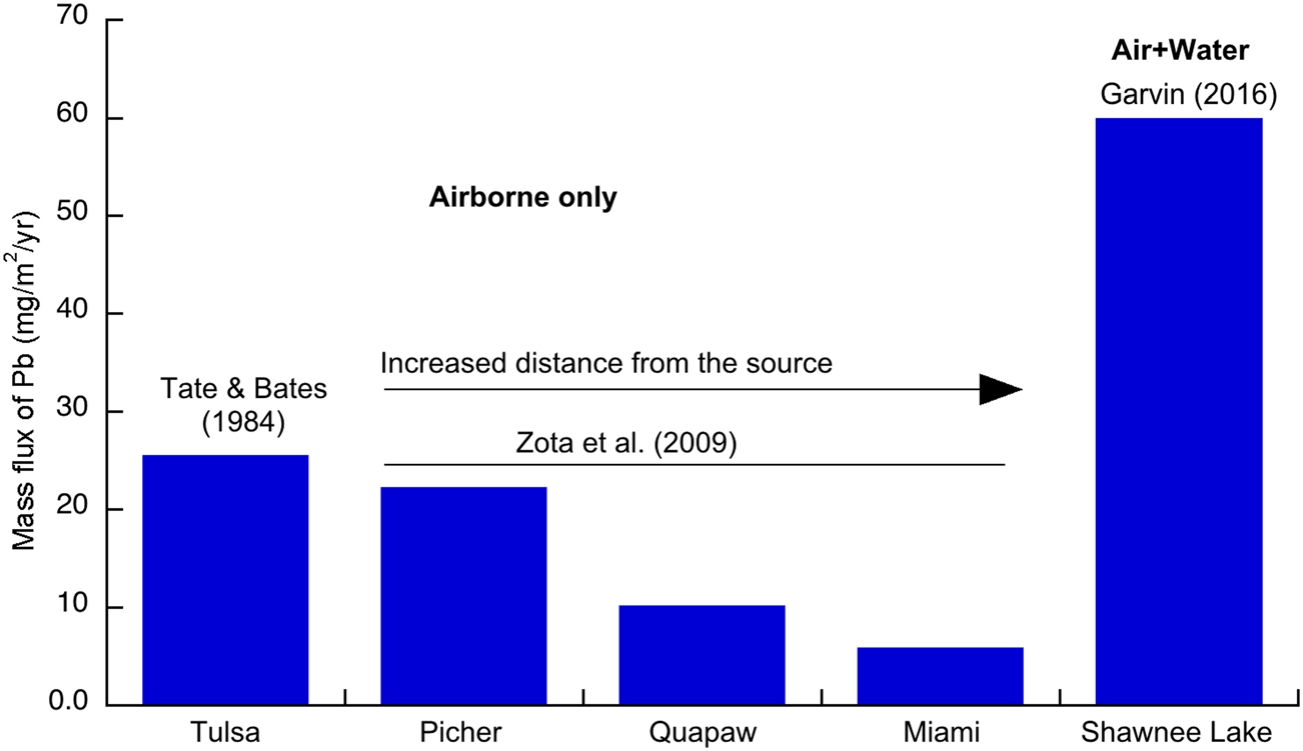
Estimated Pb mass flux in Tulsa and locations affected by chat piles in Oklahoma. Picher, Quapaw, and Miami are located <1, 5, and 18 km south of the chat piles, respectively, and Tulsa is about 150 km southwest of Picher.

Assuming the airborne Pb is only associated with fine particles of PM_10_, we estimated that the mass flux of airborne Pb in Picher, Quapaw, and Miami was 22.2, 10.2 and 5.9 mg/m^2^/year, respectively (Figure 6). The estimated mass flux of airborne Pb in Picher is close to the Pb flux in Tulsa measured in the early 1980s (Tate & Bates, 1984) (Figure 7). Since both Miami and the Shawnee Lake (Garvin, 2016) are located approximately 18 km south of the chat piles, we therefore substituted the mass flux in Miami for the Shawnee Lake and estimated that for the Shawnee Lake sediment, the contribution of airborne Pb to the overall Pb influx is about 9.75%. It is likely that the contribution of airborne Pb to the aquatic environments closer to the chat piles is substantially higher with the increase of airborne mass flux of Pb.

## 4 Discussion

It is not surprising that chat piles contain higher concentrations of Pb, possibly Zn and Cd, especially in the fine‐sized material, relative to background soils and human health risk levels. Results of our study further highlight that a noticeable portion of these fine particles have diameters around 75 μm, which typically have the lowest threshold friction velocity of aeolian transport, therefore may be strongly subject to wind erosion and subsequent transport and deposition (Shao, 2000). Once airborne, these fine, high‐Pb‐content particles may be inhaled by humans or deposited in the soil and water environment many kilometers away from the chat pile source area (Field et al., 2010; Ravi et al., 2011). The erodibility of the chat pile particles, however, may be reduced during the rainy seasons when the moisture content of the chat materials is high (Figure 6). This observation is well in line with the erodibility of soil particles with different moisture content measured in the field and laboratory studies (Ravi et al., 2011). It should also be noted that a predominant direction of Pb‐laden particle deposition is unlikely as the TSMD area has fairly diverse winds despite the frequently occurred wind from the south in spring (Figure 3).

Results of our study also show that although the concentrations of airborne PM in Picher and Tulsa are similar, the concentrations of airborne Pb in Picher are substantially higher than those of Tulsa (Figures 4 and 5), despite the latter being the primary metropolitan area in northeastern Oklahoma. Moreover, Picher was largely void of industrial and human activities since 2010 as it was officially disincorporated on 1 September 2009. This observation speaks strongly that the PM in Picher contains disproportional high concentrations of Pb compared to PM generated in normal urban areas.

Not all airborne Pb in northeastern Oklahoma and the surrounding TSMD area, however, is directly sourced from the chat piles. Zota et al. (2009) showed that mine waste source contributed nearly 40% of the PM_10_‐PM_2.5_ mass in the Picher area, and this contribution gradually decreased further away from the center of the chat piles. Hu et al. (2007) reported that some two thirds of the nearly 300 original chat piles have been partially excavated, with the wastes used as road gravel in the district and surrounding areas. Our own observation also confirmed that chats were widely used as fill in the yards of residences and school playgrounds. These operations could raise additional health concern as Pb‐laden dust may be emitted from paved and unpaved roads, and the ongoing removal and processing of chat for construction projects may all contribute to the finer, more respirable particles. To the best of our knowledge, the flux of Pb‐laden PM from these diverse sources (e.g., paved and unpaved roads) has not been measured in the Picher area. On the Colorado Plateau, USA, a study showed that areas adjacent to the off‐highway vehicle area could generate many times more dust than areas without vehicle disturbance (Nauman et al., 2018).

Due to the lack of on‐site monitoring, the direct estimate of atmospheric deposition of Pb to the soil and water environments in the TSMD is currently unknown, although many studies have confirmed that Pb in the soil and water in this area is substantially higher than the background level (e.g., Beattie et al., 2017; Garvin, 2016; Potra et al., 2017). By synthesizing a variety of existing studies, we estimated that about 10% of the accumulated Pb in the Shawnee Lake sediment in northeastern Oklahoma may be sourced from airborne pathways. This is likely a conservative estimate because the input of airborne Pb into the aquatic system may be also contributed by particles coarser than PM_10_, which were not counted in the Zota et al. (2009) study. Additionally, our study only considered the direct vertical inputs of airborne Pb, and part of the Pb‐contain dust may be filtered by plant canopies and subsequently rained out and incorporated in runoff. Given the fact that the magnitude of mass flux of airborne Pb is directly related to the distance to the chat pile source area, we argued that the contribution of airborne Pb to the aquatic systems closer to the chat piles is substantially higher than that of the Shawnee Lake.

**Figure 8:**
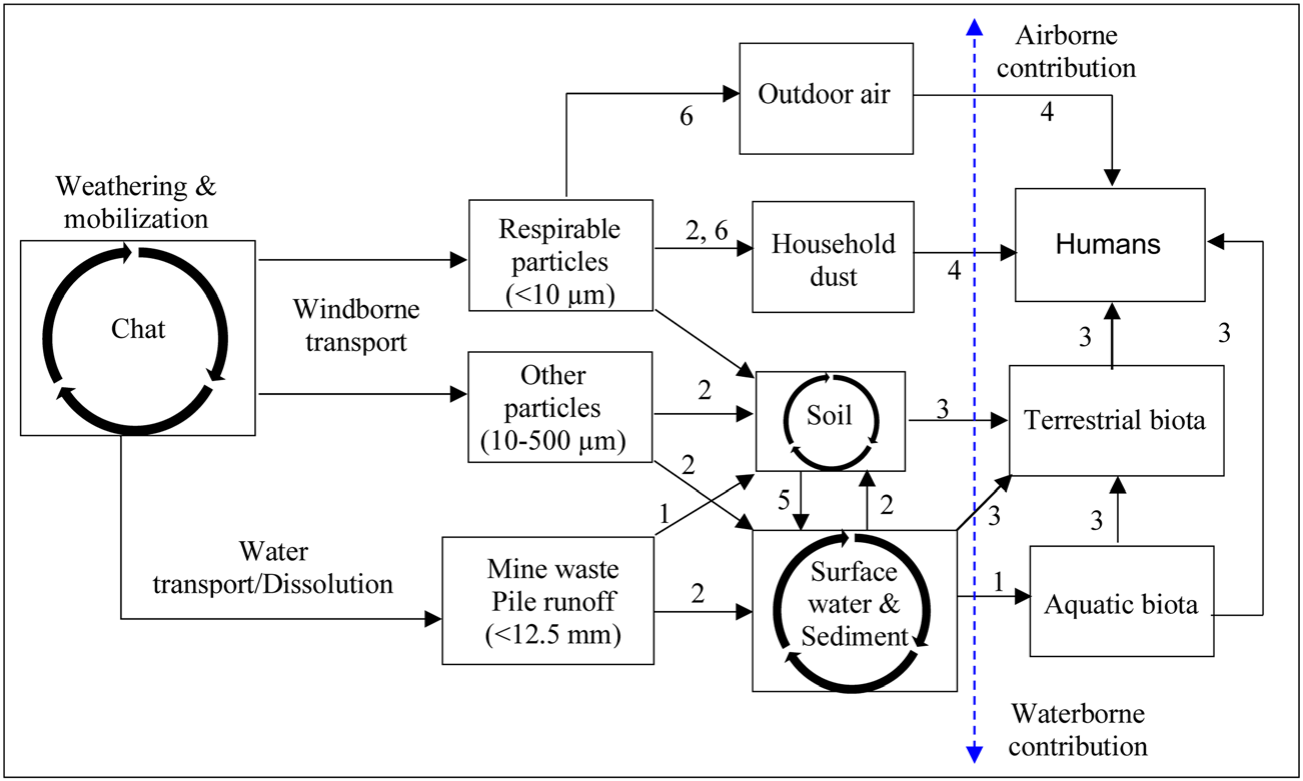
A conceptual model illustrating the fate and transport of Pb from the chat piles via waterborne and windborne processes to humans, terrestrial, and aquatic biota. The processes indicated by the numbers are 1. Adsorption, 2. Deposition, 3. Ingestion, 4. Inhalation, 5. Runoff, 6. Suspension. The blue double‐arrowed line shows the direction of the increased importance of airborne and waterborne contributions of Pb. Modified from Schaider et al. (2007).

The observed distribution of PM and airborne Pb, in combination with their transport pathways and contributions to aquatic environments, enabled us to develop a conceptual model illustrating the fate and transport of Pb from the chat piles (Figure 8). In this model, original mining rocks went through various extent of physical and biogeochemical weathering processes to yield chat piles with various particle size ranges and secondary minerals. Subsequent transport of the material, depending on particle size, solubility of the minerals, and location, may be initiated by fluvial (waterborne) and (or) aeolian (airborne) processes. Particles smaller than 75 μm can be transported hundreds of meters or even regionally by wind via the mechanism of dust emission (Field et al., 2010). Among the windborne particles, smaller particles are also more relevant to inhalation exposure (<10 μm) and rates of ingestion via hand‐mouth contact with contaminated soils and household items (Duggan et al., 1985) and metal absorption from ingested particles (Barltrop & Meek, 1979). The contribution of Pb to human bodies via airborne inhalation versus ingestion of food and drinks, however, is still unknown.

Our study has some known limitations. Although we detected that a substantial proportion of chat pile particles may be subject to different modes of wind transport, we did not directly measure the magnitude and variation of the wind transport nor did we simulate the dispersion of windborne fine particles. We also recognize that the data used in this study were derived from different sources and certain quality control measures may be desirable. Lastly, the estimate of airborne Pb contribution to the net accumulation of Pb in the lake sediment is simplified without considering Pb that may be consumed by the aquatic life, dissolved Pb in the lake water, and even Pb that may flow out of the lake. Nevertheless, the results of our study provide quantitative evidence to support the claim that the issue of windborne transport may be important (Hu et al., 2007). Given the fact that soil and dust ingestion may be particularly important in children, it is thus imperative to obtain in‐depth understanding of the underlying geochemical cycling that leads to these potentially adverse health impacts.

## 5 Conclusions

We found that chat piles in the Picher mining area in northeastern Oklahoma contain ~20% and 6% of fine particles that are subject to windborne transport and human inhalation, respectively, and these particles contain disproportionally high concentrations of Pb. Airborne Pb in Picher is the highest in January and is notably affected by precipitation. Airborne Pb in Picher is 2–5 times higher than that of Tulsa, a major metro area in northeastern Oklahoma. We estimated that airborne Pb may contribute up to 10% of annual Pb mass flux to a lake 18 km away from the chat piles in Picher and probably a much higher contribution for soil and water located adjacent to Picher. These discoveries have important implications in how to manage the risk of Pb exposure to human and aquatic biota, as well as guide remediation efforts in this region. Despite known limitations, our study represents the first attempt to evaluate the significance of Pb‐laden airborne particulate matter from a large abandoned mining area where the humans are particularly vulnerable to metal exposure. While this study only focused on Pb, future research should also examine the concurrent exposure to the combination of metals, which is a better reflection of the “real world,” and may have synergistic neurotoxic effects on humans.

## Conflict of Interest

The authors declare no conflicts of interest relevant to this study. Any use of trade names is for descriptive purposes only and does not imply endorsement of any format.

## Data Availability

The airborne particulate matter and Pb data were downloaded from the U.S. Environmental Protection Agency Air Quality System (AQS) database (https://www.epa.gov/outdoor-air-quality-data/interactive-map-air-quality-monitors).
